# The Ultra-Potent and Selective TLR8 Agonist VTX-294 Activates Human Newborn and Adult Leukocytes

**DOI:** 10.1371/journal.pone.0058164

**Published:** 2013-03-04

**Authors:** David J. Dowling, Zhen Tan, Zofia M. Prokopowicz, Christine D. Palmer, Maura-Ann H. Matthews, Gregory N. Dietsch, Robert M. Hershberg, Ofer Levy

**Affiliations:** 1 Department of Medicine, Division of Infectious Diseases, Boston Children’s Hospital, Boston, Massachusetts, United States of America; 2 Harvard Medical School, Boston, Massachusetts, United States of America; 3 Division of Pediatrics, Xin Hua Hospital Affiliated to Shanghai Jiao Tong University School of Medicine, Shanghai, China; 4 VentiRx Pharmaceuticals, Inc., Seattle, Washington, United States of America; University Hospital Zurich, Switzerland

## Abstract

**Background:**

Newborns display distinct immune responses that contribute to susceptibility to infection and reduced vaccine responses. Toll-like receptor (TLR) agonists may serve as vaccine adjuvants, when given individually or in combination, but responses of neonatal leukocytes to many TLR agonists are diminished. TLR8 agonists are more effective than other TLR agonists in activating human neonatal leukocytes in vitro, but little is known about whether different TLR8 agonists may distinctly activate neonatal leukocytes. We characterized the in vitro immuno-stimulatory activities of a novel benzazepine TLR8 agonist, VTX-294, in comparison to imidazoquinolines that activate TLR8 (R-848; (TLR7/8) CL075; (TLR8/7)), with respect to activation of human newborn and adult leukocytes. Effects of VTX-294 and R-848 in combination with monophosphoryl lipid A (MPLA; TLR4) were also assessed.

**Methods:**

TLR agonist specificity was assessed using TLR-transfected HEK293 cells expressing a NF-κB reporter gene. TLR agonist-induced cytokine production was measured in human newborn cord and adult peripheral blood using ELISA and multiplex assays. Newborn and adult monocytes were differentiated into monocyte-derived dendritic cells (MoDCs) and TLR agonist-induced activation assessed by cytokine production (ELISA) and co-stimulatory molecule expression (flow cytometry).

**Results:**

VTX-294 was ∼100x more active on TLR8- than TLR7-transfected HEK cells (EC_50_, ∼50 nM vs. ∼5700 nM). VTX-294-induced TNF and IL-1β production were comparable in newborn cord and adult peripheral blood, while VTX-294 was ∼ 1 log more potent in inducing TNF and IL-1β production than MPLA, R848 or CL075. Combination of VTX-294 and MPLA induced greater blood TNF and IL-1β responses than combination of R-848 and MPLA. VTX-294 also potently induced expression of cytokines and co-stimulatory molecules HLA-DR and CD86 in human newborn MoDCs.

**Conclusions:**

VTX-294 is a novel ultra-potent TLR8 agonist that activates newborn and adult leukocytes and is a candidate vaccine adjuvant in both early life and adulthood.

## Introduction

Newborns display distinct immune responses that leave them vulnerable to higher rates of infections as compared to older children and adults [1], with over 2,000,000 deaths per year worldwide due to infection in those less than 6 months of age [2]. In particular, neonatal deficiencies of innate cellular immune responses include a decreased production of interferons, IL-12p70, TNF and other proinflammatory/Th1-polarizing cytokines [3]. Divergent neonatal immune responses also pose a challenge to efforts to enhance early life immunization, which is a key global health strategy [4]. Lack of effective immunization strategies in the neonatal period (first 28 days of life) leads to a window of susceptibility to infections in newborns and infants lasting several months. For the past decade, there has been an ongoing focus on understanding the mechanisms by which the immune system of very young is distinct from adults, particularly in response to immunization [5]. New insights into the ontogeny of innate immunity are informing development of age-specific adjuvanted vaccine formulations [6], [7], [8], with safety and efficacy remaining central priorities [9]. The coming decade is likely to focus on developing technologies that overcome or circumvent these immunological obstacles to the development of more effective early life vaccines [2].

A promising approach to enhancing immunity of newborns and infants is to use TLR agonists as novel stand-alone immunomodulators and/or as adjuvants in vaccine design [10]. Indeed, TLR agonists have been unknowingly used for decades in vaccines such as *Haemophilus influenzae* type b (HIb) vaccine containing Neisseria-derived outer membrane proteins (TLR2), Bacille Calmette-Guérin (*Mycobacterium bovis*; TLR 2, 4 and 8) [11], and certain hepatitis B vaccines that contain lipid A (TLR4) [12]. First generation vaccines, including those consisting of inactivated or attenuated virus, contained inherent TLR7 and/or TLR8 adjuvant activity [13]. Both the inactivated polio and Japanese Encephalitis vaccines, which were introduced in 1955 and 1968, respectively, contained single stranded RNA (ssRNA) TLR7/8 agonists. Additionally, the highly effective live attenuated yellow fever vaccine 17D (YF-17D) activates multiple dendritic cell (DC) subsets via TLR2, TLR7, TLR8, and TLR9 [14] and exhibits activity in children [15].

Neonatal whole blood, cord blood mononuclear cells (CBMCs) and monocytes demonstrate impaired TNF responses to agonists of TLR1–7, most notability for TLR2/6 and TLR4 activation [16]. In contrast, TLR8 agonists may possess unique activity in the very young [6], [17], [18], [19]. The TLR 7, -8 and -9 subfamily is endosomal and activated by nucleic acids [20], [21]. Single stranded viral RNAs, such as those of influenza and human immunodeficiency viruses, are natural agonists for TLR7 and TLR8 [22]. Both human dermal and myeloid DC, as well as T cells, express TLR8 [13], [23]. TLR8 agonists, including R848, induced adult-level TNF and IL-12p40/70 production in neonatal WB and CBMCs [18]. Additionally, TLR8 (including dual TLR7/8) agonists, such as those of the synthetic anti-viral imidazoquinoline (IMQ) family [24] and ssRNAs, activate robust immune responses in newborn cells relative to other TLR agonists by a mechanism refractory to the endogenous suppressive factors, such as adenosine, present in neonatal blood plasma [25], [26], [27]. However, the potential of the TLR8 pathway, including possible differences in immunologic activity between different TLR8 agonists, is incompletely characterized [28].

VentiRx Pharmaceuticals (Seattle, WA) has produced a series of novel low molecular weight (400 – 475 Da) synthetic benzazepine derivatives that selectively activate cells via TLR8. Due to its ability to re-orient immune responses towards Th1 immunity, the TLR8 agonist VTX-1463 is in development for the treatment of allergic rhinitis and has shown significant symptom reduction in atopic individuals [29]. The TLR8 agonist VTX-2337 activates monocytes, DCs and NK cells, induces IFN-γ production and increased cytolytic activity, and is being developed as an immunotherapy in multiple oncology indications [30]. In the present study, we assessed novel VentiRx benzazepine compounds (VTXs), characterized their specificity using TLR-transfected human embryonic kidney (HEK) cells, and have evaluated their bioactivity towards human newborn and adult primary leukocytes. We find that VTX-294 is an ultra-potent TLR8-selective agonist with greater potency and activity than IMQs in both neonatal and adult leukocytes and MoDCs, suggesting that it may be a particularly attractive candidate as an adjuvant towards neonatal leukocytes.

## Materials and Methods

### Ethics Statement

Non-identifiable cord blood samples were taken with approval from the Ethics Committee of The Brigham & Women’s Hospital, Boston, MA (protocol number 2000-P-000117). All blood samples from adult patients included in the experiments provided written informed consent with approval from the Ethics Committee of Boston Children’s Hospital, Boston, MA (protocol number X07-05-0223). All experiments were performed in accordance with relevant institutional and national guidelines, regulations and approvals.

### TLR Agonists and Assay Reagents

**Figure 1:**
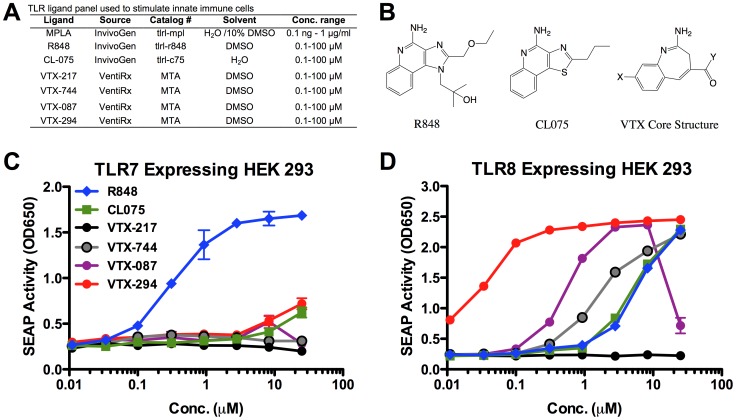
VTX-294 activates the NF-κB pathway via TLR8. (A) Seven TLR agonists were compared. (B) Representative structures of the TLR7/8 agonists used in this study. HEK-293 cells transfected with (C) human TLR7 and (D) TLR8 and an NF-κB-driven reporter SEAP gene were stimulated for 18–24 h with TLR agonists. The y-axis shows the level of SEAP activity in the Quanti-blue™ assay optical density (OD). The x-axis shows the concentration of each compound in µM. Each data point represents the mean ± SD of OD at 650 nm of triplicate culture wells. VTX-217 (black) is a structurally matched negative control for VTX-744 (gray), VTX-087 (purple) and VTX-294 (red). The IMQ benchmarks R848 and CL075 are denoted in blue and green, respectively.

The following commercially-available TLR agonists were used at the concentrations noted in the figure legends: MPLA (TLR4), R848 (TLR7/8), CL075 (TLR8/7) (InvivoGen, San Diego, CA). VTX-217 (a negative control with a similar core structure to other VTX compounds but with modifications to make inactive towards TLRs 7/8), VTX-744 (TLR8), VTX-087 (TLR8) and VTX-294 (TLR8) (VentiRx Pharmaceuticals, Inc., Seattle, WA, USA) were produced using a method of synthesis of substituted benzazepine derivatives [31]. TLR agonists (other than MPLA) were verified to be free of endotoxin (<1 EU/ml) as measured by the *Limulus amoebocyte lysate* (LAL) assay per the manufacturer’s instructions (Charles River, Wilmington, MA). TLR agonists were prepared in DMSO (Sigma-Aldrich, St. Louis, MO) as outlined in Figure 1A, and DMSO controls employed to control for any potential vehicle effects on cell activation.

### Human TLR Expressing HEK293 Cell Assays

Human embryonic kidney cells (HEK293) expressing TLR2, TLR3, TLR4, TLR5, TLR7, TLR8, or TLR9 were purchased from InvivoGen (San Diego, CA) and cultured in DMEM (Cambrex, Walkersville, MD) containing 4.5 g/L L-glucose (Sigma–Aldrich, St. Louis, MO) and 10% fetal bovine serum (FBS) (Gibco). The activity of specific TLR agonists was assessed using the secretory embryonic alkaline phosphatase (SEAP) reporter gene that is linked to NF-κB activation in response to TLR stimulation. Measurement of SEAP activity was determined using the Quanti-blue™ substrate (InvivoGen) after TLR agonist treatment for 18–24 hours using a Victor 3V 1420 Multilabel Counter (Perkin Elmer) to measure OD at 650 nM.

### Human Blood

Peripheral blood was collected from healthy adult volunteers, while human newborn cord blood was collected immediately after Cesarean section delivery of the placenta. Births to HIV-positive mothers were excluded. Human experimentation guidelines of the U.S. Department of Health and Human Services, The Brigham & Women’s Hospital, and Boston Children’s Hospital were observed, following protocols approved by the local institutional review boards. Human blood was anti-coagulated with 15–20 units/ml pyrogen-free sodium heparin (American Pharmaceutical Partners, Inc., Schaumberg, IL). All blood products were kept at room temperature and processed within 4 hrs of collection.

### Blood Sample Processing and *in vitro* Stimulation

For assessment of TLR agonist activity in whole blood, we used an adaptation of the method of Kollmann *et al*. [32], [33]. Neonatal cord blood or adult whole blood (WB) was mixed 1∶1 with sterile pre-warmed (37°C) RPMI 1640 medium (Invitrogen, Carlsbad, CA) and 180 µL of the 1∶1 suspension was added to each well of a 96 well U-bottom plate (Becton Dickinson, Franklin Lakes, NJ, USA) containing 20 µl freshly prepared specific TLR agonists at 10x the final concentration. Suspensions containing 200 µl/well were gently mixed by pipetting and incubated for 2–24 h at 37°C in a humidified incubator at 5% CO_2_. After culture, plates were centrifuged at 500×g and 110–150 µl of supernatant was carefully removed by pipetting without disturbing the cell pellet. Supernatants derived from human leukocyte stimulations were assayed by ELISA for TNF (BD Biosciences, San Jose, CA, USA) and IL-1β (eBiosciences, San Diego, CA). Additionally, WB supernatants were analysed by Multi-Analyte Profiling (MAP). Briefly, supernatant samples were frozen at −80°C and submitted to Myriad RBM (Austin, TX, USA) for screening with a modified Human InflammationMAP® v. 1.6 antigen panel, which analyzed the samples for the presence of 98 analytes associated with inflammation. The minimum threshold for each analyte was set at the minimum detectable dose for that particular assay (defined as three standard deviations above the mean background).

### Monocyte-derived Dendritic Cells (MoDCs)

Heparinized human newborn cord blood or adult peripheral blood was layered onto Ficoll-Hypaque gradients (Ficoll-Paque PREMIUM, GE Healthcare, Waukesha, WI) to collect CBMC or peripheral blood mononuclear cell layers (PBMC), respectively. Monocytes were isolated from mononuclear cell fractions by positive selection by magnetic microbeads according to the manufacturer’s instructions (Miltenyi Biotec, Auburn, CA) using CD14 or CD33 as a pan-marker for monocytes [34]. Monocyte preparations were >95% pure as assessed by flow cytometry for CD14 as previously described [25]. Isolated monocytes were cultured in tissue culture dishes at 10^6^ cells/ml in RPMI 1640 media containing fresh 10% autologous plasma, supplemented with rhIL-4 (50 ng/ml) and rhGM-CSF (100 ng/ml) (R&D Systems, Minneapolis, MN) with one additional supplement of fresh media and cytokines at day 3 of culture. After 5–6 days, immature MoDCs (HLA-DR^+^, CD14^−^, DC-SIGN^+^ as measured by flow cytometry) of >90% purity were harvested by gently pipetting only the loosely adherent fraction and re-plated (10^5^ cells/well) in 96-well U-bottom plates in the presence or absence of TLR agonists.

### Flow Cytometry

Following TLR agonist stimulation, MoDCs were resuspended in staining buffer (1×PBS, 0.5% human serum albumin (HSA)) and stained for 30 min at 4°C in the dark (1.5×10^5^/per staining) with fluorophore-labeled antibodies (FITC/HLA-DR and PE/CD86) (BD Biosciences). Cells were then centrifuged, washed, fixed (1% paraformaldehyde (PFA)) and filtered prior to flow cytometry analysis using a MoFlo Legacy cytometer (DakoCytomation, Fort Collins, CO) and analyzed using FlowJo software (Tree Star). MFI fold change was plotted after subtraction of the MFI of equivalent samples stained with an isotype control antibody.

### Statistical Analyses and Graphics

Data were analyzed using Prism for MacIntosh v. 5.0b (GraphPad Software Inc., San Diego, CA). Data in figures represent means ± SEM. p values <0.05 were considered significant. Normalcy was tested using the Shapiro-Wilk normality test. For normal sample sets, two-tailed t-test or One-way ANOVA with Bonferroni post-test were applied as appropriate. Non-normal sample sets were analyzed by Kruskal-Wallis test with Dunn’s multiple comparison post-test, by Wilcoxon signed-rank test or by Mann-Whitney test, as appropriate.

## Results

### Specificity of VTX Agonists for Human TLR7 and TLR8

To assess the selectivity of novel VTX benzazepine compounds, we characterized VTX-induced NF-κB activation in HEK-293 cells expressing human TLR7 or TLR8 in comparison to the imidazoquinolines R848 (TLR7/8) and CL075 (TLR8) Figure 1 (C–D). As expected, R848 induced NF-κB activation in both TLR7- and TLR8-transfected HEK293 cells. CL075 showed greater activation of NF-κB in TLR8- compared to TLR7-transfected cells at the highest concentration tested. Similarly, the three VTX compounds VTX-744, VTX-087 and VTX-294 demonstrated activation of NF-κB in TLR8 transfected cells, with VTX-294 also modestly activating TLR7 transfected cells at the highest concentration tested. The potency of the VTX compounds tested was assessed by comparing EC_50_ the concentration of agonist at which half maximal activation of NF-κB was observed in TLR8-transfected cells. This analysis indicated the following rank of potencies: VTX-294 (EC_50_ 0.05 µM)>VTX-087 (0.54 µM)>VTX-744 (1.68 µM)>CL075 (4.57 µM)>R848 (5.12 µM; Table S1). The structurally similar negative control compound, VTX-217, did not induce activation of NF-κB in either TLR7- or TLR8-transfected cells. Additionally, none of the VTX agonists tested activated TLR2 (the receptor for lipo*-*peptides), TLR3 (double-stranded RNA), TLR4 (lipopolysaccharide (LPS)), TLR5 (bacterial flagellum) or TLR9 (unmethylated CpG dinucleotide-containing DNA) (Figure S1).

### The Novel TLR Agonist VTX-294 Potently Activates Human Newborn and Adult Leukocytes

**Figure 2:**
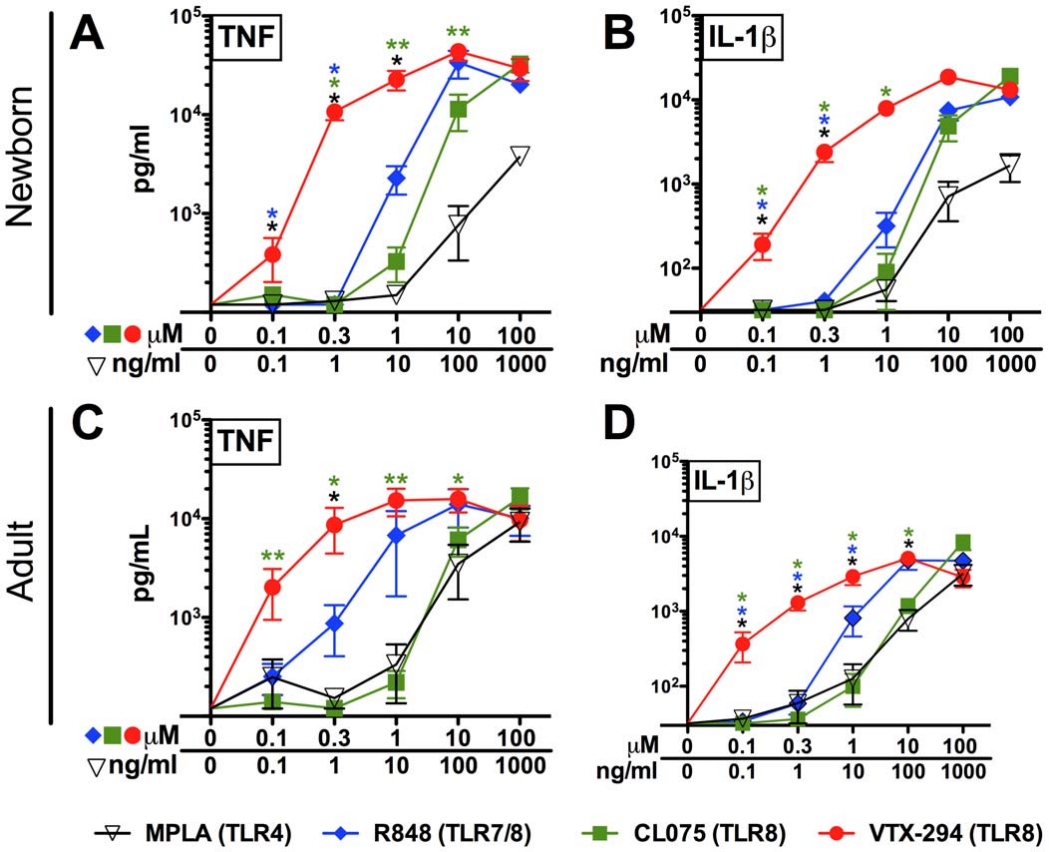
VTX-294 is more potent and effective than R-848 in inducing TNF and IL-1β in whole blood. Human neonatal and adult blood was cultured for 6 h with MPLA (TLR4), R848 (TLR7/8), CL075 (TLR8) and VTX-294 (TLR8) and supernatants collected for TNF or IL-1β ELISA. Compound concentrations are shown in µM, except for MPLA, which are shown in ng/ml. Data are shown as mean ± SEM of n = 5–8. For between-group analyses, Wilcoxon signed-rank test was applied to compare VTX-294 median value to the other compounds at a given concentration. Statistical significance is denoted as follows: *p<0.05 and **p<0.01, with black star(s) for comparison to MPLA, blue star(s) for comparison to R848 and green star(s) for comparison to CL075.

We tested the ability of VTX agonists to induce dose dependent cytokine production in human neonatal and adult blood (Figure 2). In line with the HEK293 data in Figure 1 (C–D), the negative control compound VTX-217 did not demonstrate activation of either human neonatal cord or adult peripheral blood (data not shown). As VTX-294 was the most potent of all three VTX compounds, we tested the dose-dependent cytokine induction of VTX-294 compared with MPLA, a TLR4 agonist whose congeners are adjuvant components of vaccines licensed by both the U.S. Food and Drug Administration (FDA) and European Medicines Agency (EMA), and two imidazoquinoline TLR agonist adjuvants (Figure 2). VTX-294 activated neonatal and adult blood in a dose-dependent manner, significantly inducing production of TNF and IL-1β over baseline (p<0.001). In both newborn cord and adult peripheral blood, MPLA (TLR4), R848 (TLR7/8) and CL075 (TLR8) significantly induced production of TNF and IL-1β, in a dose dependent manner (Figure 2). When compared to R848 at 0.1 µM, VTX-294 demonstrated greater efficacy, stimulating greater TNF and IL-1β production in neonatal blood (p<0.05). VTX-294 was also more potent than R848 and CL075 in inducing IL-1β production in adult WB (p<0.05). Greater potency of VTX-294 as compared to R848 and CL075 was also noted for adult TNF production, but did not reach significance (p = 0.054). Overall, VTX-294 demonstrated the greatest potency (i.e. lowest EC_50_ values) of all agonists tested for both TNF and IL-1β production in newborn cord and adult peripheral blood (Table S1). With respect to efficacy of inducing cytokine production, the maximum cytokine levels produced at higher agonist concentrations of VTX-294 (e.g., 100 µM) was similar to those induced by R848 and CL075 (Table S2).

### Comparison of TLR Agonist-induced TNF and IL-1β Production in Newborn and Adult Blood

**Figure 3:**
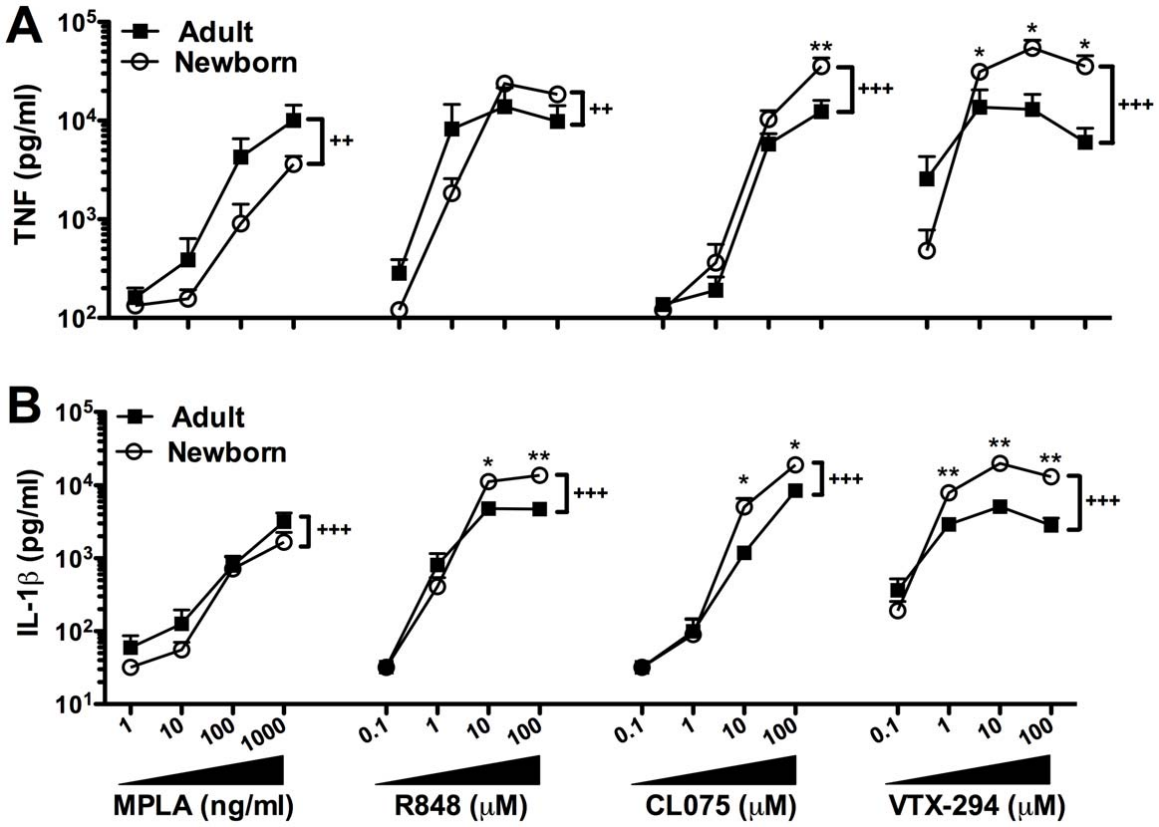
VTX 294 induces greater cytokine responses in newborn cord than adult peripheral blood. Human neonatal and adult blood was cultured for 6 h with MPLA (1, 10, 100, 1000 ng/ml), CL075, R848 and VTX-294 (all 0.1, 1, 10, 100 µM) and supernatants collected for TNF or IL-1β ELISA. Mean ± SEM of agonist-induced cytokine production are shown for n = 5–6. For comparisons between overall groups (newborn vs. adult), Kruskal-Wallis test with Dunn’s post test were applied, with significance denoted as ^++^p<0.01, ^+++^p<0.001. For analyses at individual doses (newborn vs. adult), unpaired Mann-Whitney test was applied at each concentration and statistical significances are denoted as follows: *p<0.05 and **p<0.01.

Our results demonstrate an impaired newborn WB TNF response to MPLA by between group comparison (newborn vs. adult) (Figure 3 A, p<0.01). In line with previous findings on the unique efficacy of TLR8 agonists in newborn blood [18], we demonstrated a preservation of responses to R848 and CL075, both inducing TNF production in newborn cord blood at concentrations equal to or greater than that in adult blood (p<0.01 and p<0.001 respectively) by between group comparison (newborn vs. adult). VTX-294 not only induced greater concentrations of TNF in newborn WB compared to adults (p<0.001), but also did so at individual doses (newborn vs. adult) as low as 1 µM (p<0.05). In contrast, R848 was not more effective at inducing TNF in newborn relative to adult WB, while CL075 demonstrated greater efficacy at inducing TNF at 100 µM than VTX-294. IL-1β production followed a similar pattern, with VTX-294 inducing a greater concentration of IL-1β in newborn cord blood compared to adult peripheral blood at concentrations of >1 µM (p<0.01). CL075- and R848-induced IL-1β production was significantly increased in newborn compared to adult WB at 10 µM (p<0.05) (Figure 3 B).

### Multi-Analyte Comparison of Agonist Combination-induced Cytokine Production in Newborn and Adult Blood

**Figure 4:**
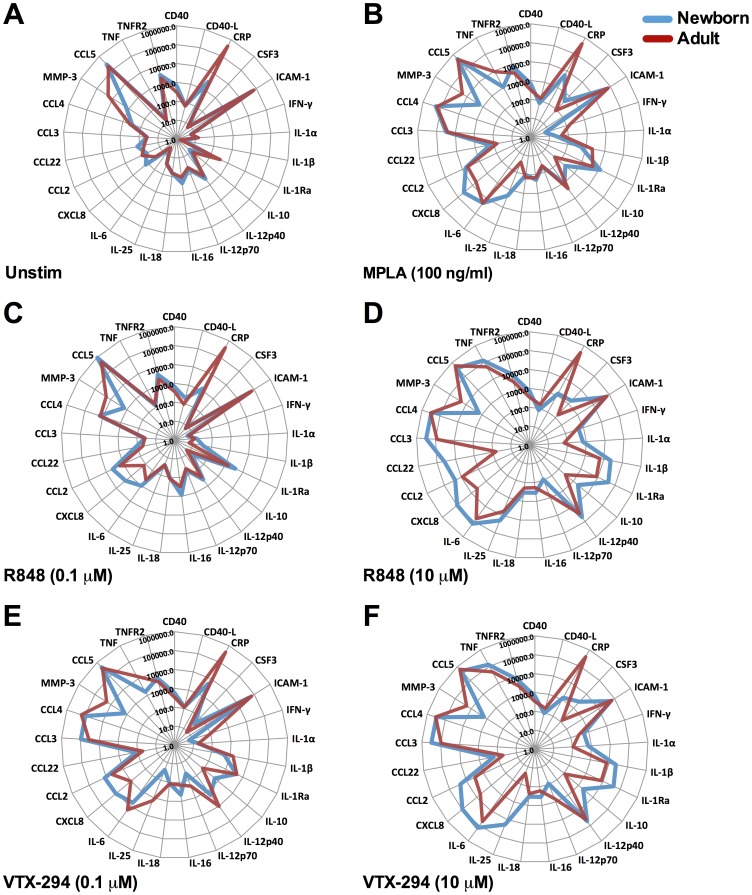
Comparison of TLR agonist-induced inflammatory biomarker production in newborn and adult blood. Human neonatal (blue) and adult (red) WB samples were cultured for 6 h with (A) vehicle (B) MPLA (TLR4), (C–D) R848 (TLR7/8) and (E–F) VTX-294 (TLR8) and supernatants recovered for analysis by Multi-Analyte Profiling (MAP), with a modified Human Inflammation MAP v. 1.6-antigen panel. Cytokine responses of 25 analytes (pg/ml) are represented in radar plots. Data are shown as mean ± SEM for n = 3.

We next employed a multiplexed bead array-based approach to provide a broader view of differences between newborn and adult TLR agonist-induced inflammatory biomarkers after 6 h of stimulation (Figure 4 and Figure S2). This approach confirmed that compared to adult blood, newborn blood contained: 1) lower basal CRP concentrations (Figure 4 A), 2) greater human growth hormone [35] (Figure S2 A), 3) increased basal CXCL-8 (IL-8) concentrations [36], and 4) greater TLR4-mediated production of IL-10 (Figure 4 B) [32]. We also confirmed that TLR7/8 agonists induce similar concentrations of IL-12p40 in both newborn and adult WB [32], although IL-12p70 production in neonates was reduced [37] (Figure 4 C–F).

Newly identified differences in newborn as compared to adult blood, included a consistent increase in both EN-RAGE (extracellular newly identified RAGE-binding protein) and myeloperoxidase (MPO) under all stimulated conditions (Figure S2). The increase in MPO may reflect the greater number of neutrophils in newborn cord blood compared to adult [3]. TLR-mediated production of oncostatin M (OSM), an IL-6 family cytokine, was greater in newborn, as compared to adult blood for MPLA (100 ng/ml), R848 (10 µM) and VTX-294 (10 µM; Figure S2). Conversely, under all conditions tested, newborn cord blood secreted lower concentrations of matrix metalloproteinase-3 (MMP-3) (Figure S2).

### VTX-294 Induces Greater Cytokine Responses than R848 in Human Neonatal Blood

**Figure 5:**
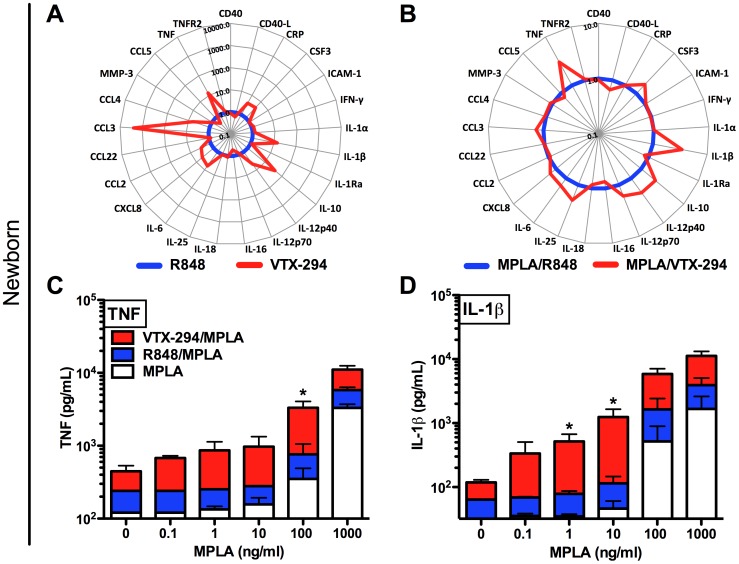
Whether tested alone or together with MPLA, VTX-294 has greater cytokine-inducing potency than R848 in human neonatal blood. Human neonatal WB samples were cultured for 6 h with VTX-294 or R848 (both 0.1 µM) and supernatants recovered for analysis by Multi-Analyte Profiling (MAP), with a modified Human Inflammation MAP v. 1.6-antigen panel. Cytokine responses of 25 analytes are represented in radar plots demonstrating fold-change of (A) 0.1 µM VTX-294 (red) over 0.1 µM R848 (blue), or (B) WB concurrently stimulated in the presence of 100 ng/ml MPLA with 0.1 µM VTX-294 (red) over 100 ng/ml MPLA with 0.1 µM R848 (blue). TNF (C) and IL-1β (D) responses in neonatal WB are shown for VTX-294 (0.1 µM), R848 (0.1 µM) and buffer control added to increasing concentrations of MPLA (0–1,000 ng/ml) are shown. Data are shown as mean ± SEM for n = 4. Statistical significance was determined using paired t-test comparing MPLA/VTX-294 treated compared to MPLA/R848 (C, D); *p<0.05.

At the lowest tested concentration (0.1 µM), VTX-294 induced greater concentrations of nearly all cytokines tested than R848 (Figure 5 A). Specifically, when expressed as fold change over R848 induction, VTX-294 induced >10 fold more TNF, IL-1β and IL-10 and >5 fold more IL-6 and IL-12p40 (Figure 5 A). Compared to R848, VTX-294 induced greater production of multiple chemokines, including CCL3 (MIP-1α; >1000 fold), CCL4 (MIP-1β), CCL-2 (MCP-1) and CXCL-8 (IL-8) (all >5 fold), CSF3 (G-CSF) and CRP (both >5 fold) (Figure 5 A).

As combinations of TLR agonists may have synergistic immune enhancing effects and several vaccine formulations employ combinations of adjuvants, we tested combinations of MPLA with either R848 or VTX-294 in newborn WB. Specifically, when expressed as fold change over MPLA/R848 induction, MPLA/VTX-294 induced >5 fold more TNF and IL-1β (Figure 5 B). We further assessed the effects of increasing concentrations of MPLA (Figure 5 C–D) with fixed concentrations of R848 and VTX-294 (both 0.1 µM) measuring these two key cytokines. We found that VTX-294 acted in synergy with MPLA, particularly at lower concentrations of the MPLA. Similarly, we characterized adult WB comparing R848 and VTX-294 (both 0.1 µM) (Figure S3 A) with or without co-addition of MPLA (Figure S3 B–D).

**Figure 6:**
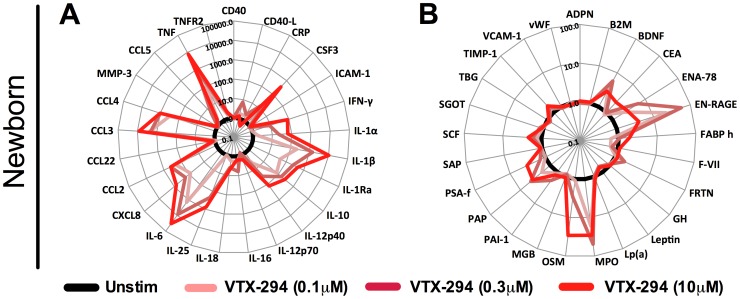
VTX-294 induces dose-dependent production of multiple soluble plasma analytes. Human neonatal WB samples were cultured for 6 h with VTX-294 (0.1–10 µM) and supernatants recovered for analysis by Multi-Analyte Profiling (MAP), with a modified Human Inflammation MAP v. 1.6-antigen panel. Responses for 50 analytes (pg/ml) are represented in radar plots showing fold-change of VTX-294 (red) over un-stimulated (black). Radar plots show (A) 25 immunomodulatory cytokines and chemokine’s, and (B) 25 hormones, growth and stress factors.

To further characterize the immunostimulatory potential of VTX-294, we assessed dose-dependent stimulation of newborn WB measuring 50 analytes, at concentrations of 0.1–10 µM (Figure 6 A–B). When graphed as fold change over unstimulated, a dose-dependent induction was clearly evident for multiple cytokines including TNF, IL-1β, IL-1α, IL-1Ra, IL-6, CXCL-8 (IL-8), G-CFS, IL-12p40 and IL-10 (Figure 6 A). The low dose of VTX-294 (0.1 µM) induced a similar cytokine profile to MPLA-mediated responses (Figure A4 A–B). In newborn whole blood, a combination of VTX-294 and MPLA enhanced production of IL-1β, IL-1α, IL-12p40, IL-10 and TNF in comparison to MPLA alone (Figure S4 C–D). VTX-294-assocaited enhancements of IL-12p70, IL-25 and IFN-γ production were selectively evident in adult whole blood.

### VTX-294 Potently Activates Human Neonatal MoDCs

**Figure 7:**
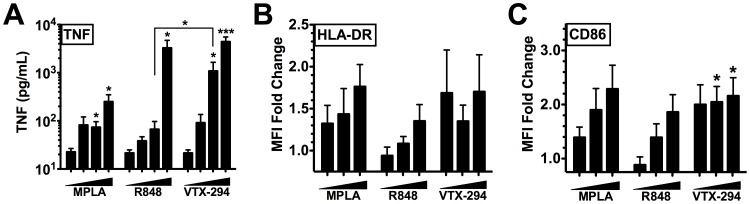
VTX-294 potently activates human neonatal MoDCs. Stimulation of neonatal MoDCs for 24 h. Supernatants collected for (A) TNF ELISA and DCs for measurement of (B) HLA-DR and (C) CD86 expression by flow cytometry. Dose dependent surface expression of co-stimulatory markers with MPLA (1, 10, 100 ng/ml), R848 (0.1, 0.3, 10 µM) and VTX-294 (0.1, 0.3, 10 µM) is shown as mean fluorescent intensity (MFI) represented as fold change over vehicle control (mean ± SEM, n = 3); *p<0.05.

As antigen-presenting cells are key targets for candidate adjuvants, we next compared dose-dependent responses of human neonatal MoDCs to MPLA (1–100 ng/ml), R848 and VTX-294 (both 0.1–10 µM) with respect to production of TNF, as well as expression of HLA-DR and the co-stimulatory molecule CD86 (Figure 7). MPLA induced dose-dependent TNF production at both 10 and 100 ng/ml (p<0.05) compared to unstimulated DCs. Whereas VTX-294 induced MoDC TNF production at concentrations as low as 0.3 µM, R848 induced significant levels of this cytokine only at 10 µM. The greater potency of VTX-294 compared to R848 in neonatal MoDCs was also observed with respect to induction of surface HLA-DR and the significantly increased CD86 expression (Figure 7 B, C).

## Discussion

Multiple licensed vaccines induce effective activation of innate immune responses by incorporating live attenuated pathogens or microbial products now known to contain TLR agonists [38]. However, with the continued development and use of inactivated subunit based vaccines, adjuvants have become increasingly important in order to overcome underdeveloped and distinct immune responses in immunocompromised populations such as newborns and infants [4]. Recent studies highlight that immunologic signatures can predict vaccine efficacy [14], suggesting that characterizing the immunostimulatory properties of candidate adjuvants may provide insight into their translational potential. We report here a broad characterization of secreted cytokine/chemokine and inflammatory proteins in a neonatal whole blood system in response to the ultra-potent and selective TLR8 Agonist VTX-294. We confirm prior studies that revealed a neonatal bias towards TLR-mediated IL-6 and IL-10 responses [6], [39] and have also identified a similar neonatal bias towards TLR-mediated CXCL-8 (IL-8) production. Characterizing the ontogeny of innate immune responses may eventually inform development of adjuvant vaccine formulations that are tailored to induce distinct immune polarization for targeted neonatal and infant vaccines. Additional distinct characteristics of neonatal innate cells, including their reduced poly-functionality - the capability of an individual cell to produce multiple cytokines simultaneously, likely contribute to age-specific responses to TLR agonists [32].

TLR agonists are by now an established class of molecules with potential vaccine adjuvant properties [40]. Indeed, the detoxified LPS MPLA is a component of several licensed vaccines including Cervarix that is approved for school-aged girls [41]. Although both LPS and MPLA are agonists of TLR4, LPS triggers toxic inflammation whereas MPLA is potently immunostimulatory, but exhibits only 0.1 to 1% of the toxicity of its parent molecule and is employed as an adjuvant in [42]. Newborns demonstrate an impaired TNF response to LPS [32], [43], [44], but to our knowledge, ours is the first study to compare MPLA-induced cytokine production in cord and adult WB, revealing an impaired ability of human neonatal blood leukocytes and MoDCs to mount inflammatory responses to this TLR agonist.

We demonstrate for the first time that VTX-294, a small benzazepine molecule that specifically and potently targets TLR8, presents a unique opportunity for augmenting innate responses in human newborns. TLR8 (including dual TLR7/8) are refractory to the inhibitory neonatal adenosine system [27] and can therefore overcome suboptimal newborn innate immune responses to induce adult-level concentrations of several key cytokines [45], [46]. We demonstrate here that VTX-294 induced key cytokines to concentrations comparable or greater than those observed in adult blood. We believe this is the first reported TLR agonist with such activity. Monocytes account for the majority of TLR-, including TLR8-, mediated TNF production in whole blood [18], [25]. Additionally, as we compared TLR agonists in blood derived form the same newborns, our data suggest that robust responses induced by VTX-294 in newborn cord blood are not due to differences in cellular composition but rather to the inherent potency and efficacy of VTX-294. VTX-294 was not only at least as effective as R848 in inducing cytokine production, but also demonstrated ∼10-fold greater potency in both WB and MoDC systems. When comparing to MPLA in newborn and adult blood, VTX-294 achieved higher maximum cytokine production for TNF, IL-10 and IL-12p40. When added to human neonatal MoDCs cultured in autologous plasma, VTX-294 was significantly more potent than R848, induced greater maximum TNF responses than MPLA. Even at the lowest concentrations tested VTX-294 also tended to enhance surface expression of HLA-DR and the co-stimulatory surface molecule CD86.

The potency of VTX-294 compared to R848 was also evident in comparing combinations of these agonists with MPLA. Combinations of TLR agonists may synergistically generate optimal cytokine production important for adjuvant activity [47]. For example, the live attenuated yellow fever vaccine YF-17D is one of the most effective vaccines available and activates multiple DC subsets via TLR2, 7, 8, and 9 [48]. Effective adjuvant systems already exist that combine MPLA and alum (AS04, developed by GlaxoSmithKline) [49] and *in vitro* studies with defined combinations of TLR agonists re-enforce the potential of such combined-adjuvant design [50], [51]. More potent adjuvants (or adjuvant combinations) may allow for antigen dose sparing or may allow for single immunization strategies. Furthermore, addition of TLR8 agonists, either in conjugated form or as a freely soluble mixture, with vaccinal antigens enhances induction of adaptive immune responses to a greater extent than do TLR4 or TLR9 agonists [52]. The impact of impaired MPLA responses in early life may be seen in a recent phase 3 trial of the RTS,S/AS01, an MPLA-adjuvanted malaria vaccine, that demonstrated reduced efficacy in African infants aged 6 to 12 weeks [53], as compared to those who were 5 to 17 months of age [54].

Overall, TLR8 agonists possess greater immunostimulatory properties than agonists of other TLRs, not only at birth (cord blood) but also in peripheral blood obtained during infancy, an important phase of susceptibility and immunization schedules [6]. Future *in vitro* studies should focus on characterizing the mechanisms underlying the potency and efficacy of VTX-294. TLR8 is preferentially localized to the early endosome and the endoplasmic reticulum but not to the late endosome or lysosome [55]. Subsequent studies should determine if the VTX compounds more effectively co-localize to these compartments. The phagosome is an efficient platform for the recognition of diverse ligands, sometimes involving co-operative interactions between TLR8 and other TLRs [56], or even other classes of PRRs [57]. Additionally, our lab has previously shown that CL075 can also activate DCs via other classes of PRRs, such as the inflammasome [17], which is supported by reports that imidazoquinolines can trigger induction of inflammatory cytokines in the absence of TLR 7 or 8 agonist activity [58]. Lastly, using molecular modeling studies, Govindaraj *et al.* demonstrated that the binding affinity of the TLR8 agonist R848 plays a key role in species-specificity [59]. Therefore, future studies to determine the binding affinity of VTX-294 in comparison with IMQs would be of interest. Future translational studies should evaluate the safety and efficacy of vaccine formulations containing TLR8 agonists in newborn animals, focusing on non-human primates that express TLR8 with structural and functional similarities to humans [60], [61].

As safety and reactogenicity will be key considerations in developing novel adjuvanted vaccines, both the immunological and toxicological properties of novel vaccine formulations should be benchmarked against currently used adjuvants such as aluminum salts, oil emulsions, MPLA and approved pediatric vaccines that contain endogenous TLR agonists, such as Bacille Calmette–Guérin [62]. Finally, novel means of localizing the action of adjuvants, as has been done with the TLR7/8 agonist 3M-052, that contains a lipid tail modification of the imidazoquinoline that serves to localize its adjuvant action in tissues and prevent systemic cytokine induction, may represent additional approaches for enhancing safety while maintaining efficacy [63].

The data presented here support the potential adjuvant activity of VTX-294 *in vitro*. Furthermore, VTX-294 activates human newborn and adult leukocytes and MoDCs with potency and efficacy that exceed several other TLR agonists, including MPLA. VTX compounds can be produced to GMP quality in quantities that allow clinical trials, and have been recently tested in a Phase I clinical trials in patients with advanced solid tumors [30]. Our results suggest that VTX compounds may have additional applications as stand-alone immunomodulators or as vaccine adjuvants that can enhance neonatal DC function.

## Supporting Information

Figure S1 **VTX agonists do not activate TLRs-2, -3, -4, or -9.** HEK-293 cells transfected with various human TLR and an NF-κB-driven reporter SEAP gene were stimulated for 18–24 h with TLR agonists. The y-axis shows the level of SEAP activity in the Quanti-blue™ assay by OD. The x-axis shows the concentration of each compound in µM with the exception of poly I:C, LPS, and flagellin which are expressed in µg/ml, ng/ml and µg/ml, respectively. Each data point represents the mean ± SD of OD at 650 nm of triplicate culture wells. The positive control compound for each TLR is indicated in black.
(TIFF)

Figure S2 **Comparison of TLR agonist-induced hormones, growth and stress factors in newborn and adult blood.** Human neonatal (blue) and adult (red) WB samples were cultured for 6 h with (A) vehicle (B) MPLA (TLR4), (C–D) R848 (TLR7/8) and (E–F) VTX-294 (TLR8) and analysis by Multi-Analyte Profiling (MAP), with a modified Human Inflammation MAP v. 1.6-antigen panel. Responses of 25 hormones, growth and stress factors (pg/ml) are represented in radar plots. Data are shown as mean ± SEM for n = 3.
(TIFF)

Figure S3 **VTX-294 induces greater cytokine responses than R848 in human adult blood.** Human adult WB samples were cultured for 6 h with VTX-294 or R848 (both 0.1 µM) and supernatants recovered for analysis by Multi-Analyte Profiling (MAP), with a modified Human Inflammation MAP v. 1.6-antigen panel. Cytokine responses of 25 analytes (pg/ml) are represented in radar plots showing fold-change of (A) VTX-294 (red) over R848 (blue) alone (both 0.1 µM), or (B) WB concurrently stimulated in the presence of 100 ng/ml MPLA with 0.1 µM VTX-294 (red) over 100 ng/ml MPLA with 0.1 µM R848 (blue). TNF (C) and IL-1β (D) responses in adult WB are shown for VTX-294 (0.1 µM), R848 (0.1 µM) and buffer control added to increasing concentrations of MPLA (0–1,000 ng/ml) are shown. Data are shown as mean ± SEM for n = 4. Statistical significance was determined using paired t-test comparing MPLA/VTX-294 treated compared to MPLA/R848 (C, D); *p<0.05.
(TIFF)

Figure S4 **Comparison of MPLA, VTX-294 and combinatory MPLA-VTX-294-induced cytokines in newborn and adult blood.** Human neonatal (blue) and adult (red) WB samples were cultured for 6 h. Cytokine responses of 25 analytes (pg/ml) are represented in radar plots showing fold-change of (A) un-stimulated vehicle to MPLA (TLR4, all 100 ng/ml), (B) un-stimulated vehicle to VTX-294 (TLR8, all 0.1 µM), (C) MPLA to VTX-294 (TLR8, all 0.1 µM) and (C) MPLA to MPLA with VTX-294, with a modified Human Inflammation MAP v. 1.6-antigen panel. Data are shown as mean ± SEM for n = 3.
(TIFF)

Table S1 (DOC)

Table S2 (DOC)
