# Supplementary figures and images for: The Ultra-Potent and Selective TLR8 Agonist VTX-294 Activates Human Newborn and Adult Leukocytes
Source: PLoS One. 2013 Mar 4;8(3):e58164. doi: 10.1371/journal.pone.0058164 (PMC3587566; doi:10.1371/journal.pone.0058164)

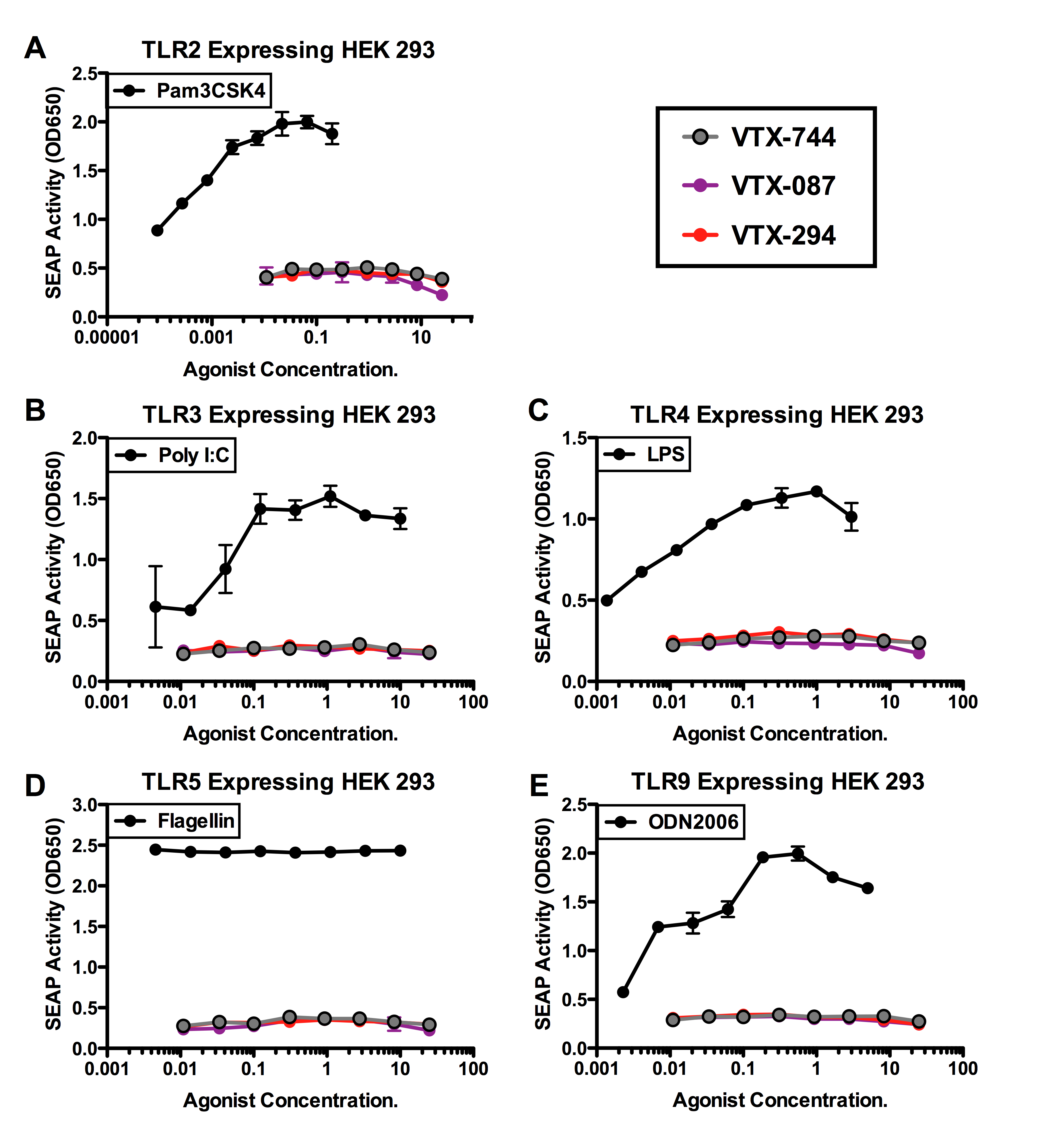

Supplement: Figure S1 — VTX agonists do not activate TLRs-2, -3, -4, or -9. HEK-293 cells transfected with various human TLR and an NF-κB-driven reporter SEAP gene were stimulated for 18–24 h with TLR agonists. The y-axis shows the level of SEAP activity in the Quanti-blue™ assay by OD. The x-axis shows the concentration of each compound in µM with the exception of poly I:C, LPS, and flagellin which are expressed in µg/ml, ng/ml and µg/ml, respectively. Each data point represents the mean ± SD of OD at 650 nm of triplicate culture wells. The positive control compound for each TLR is indicated in black. (TIFF) [file pone.0058164.s001.tiff]

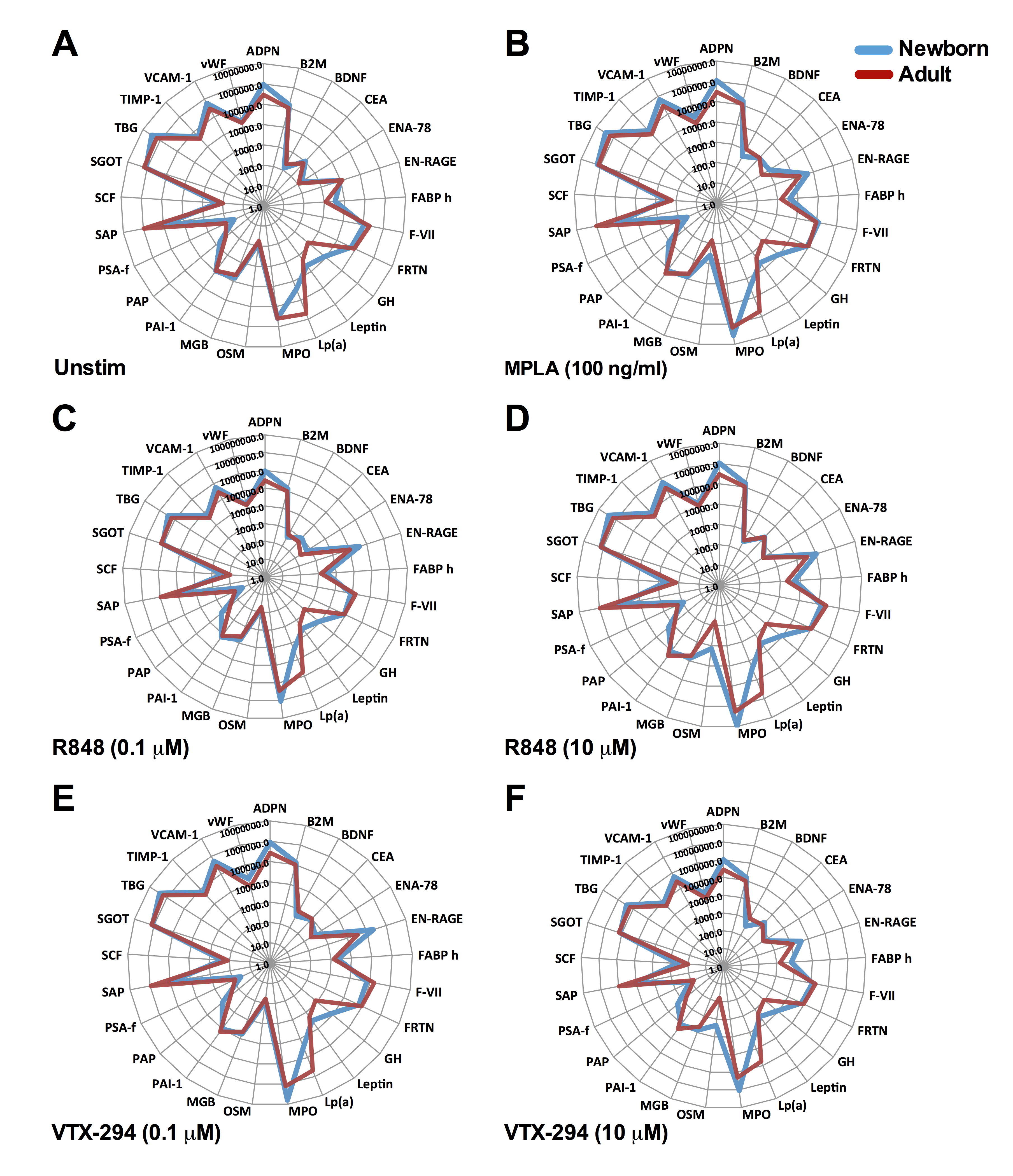

Supplement: Figure S2 — Comparison of TLR agonist-induced hormones, growth and stress factors in newborn and adult blood. Human neonatal (blue) and adult (red) WB samples were cultured for 6 h with (A) vehicle (B) MPLA (TLR4), (C–D) R848 (TLR7/8) and (E–F) VTX-294 (TLR8) and analysis by Multi-Analyte Profiling (MAP), with a modified Human Inflammation MAP v. 1.6-antigen panel. Responses of 25 hormones, growth and stress factors (pg/ml) are represented in radar plots. Data are shown as mean ± SEM for n = 3. (TIFF) [file pone.0058164.s002.tiff]

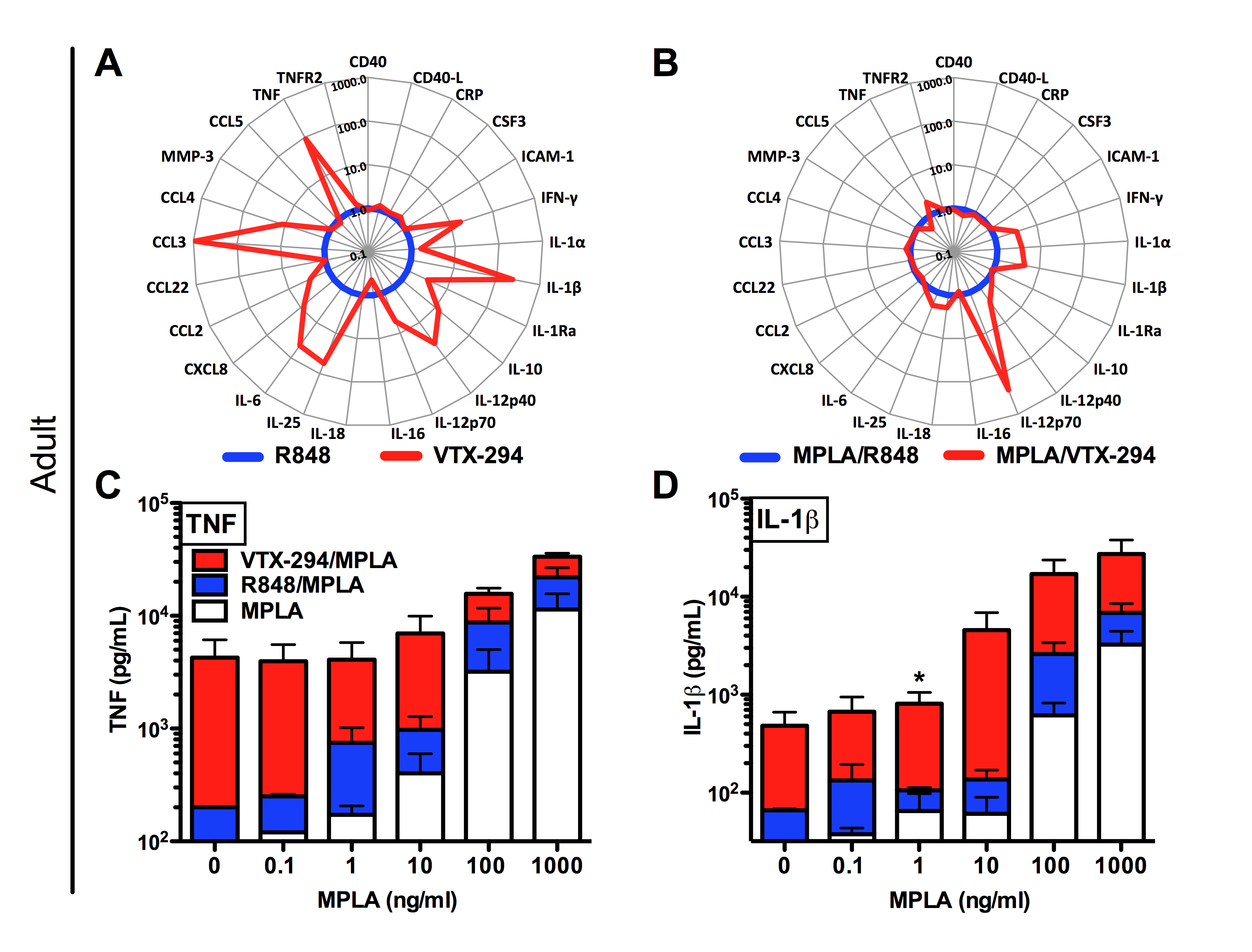

Supplement: Figure S3 — VTX-294 induces greater cytokine responses than R848 in human adult blood. Human adult WB samples were cultured for 6 h with VTX-294 or R848 (both 0.1 µM) and supernatants recovered for analysis by Multi-Analyte Profiling (MAP), with a modified Human Inflammation MAP v. 1.6-antigen panel. Cytokine responses of 25 analytes (pg/ml) are represented in radar plots showing fold-change of (A) VTX-294 (red) over R848 (blue) alone (both 0.1 µM), or (B) WB concurrently stimulated in the presence of 100 ng/ml MPLA with 0.1 µM VTX-294 (red) over 100 ng/ml MPLA with 0.1 µM R848 (blue). TNF (C) and IL-1β (D) responses in adult WB are shown for VTX-294 (0.1 µM), R848 (0.1 µM) and buffer control added to increasing concentrations of MPLA (0–1,000 ng/ml) are shown. Data are shown as mean ± SEM for n = 4. Statistical significance was determined using paired t-test comparing MPLA/VTX-294 treated compared to MPLA/R848 (C, D); *p<0.05. (TIFF) [file pone.0058164.s003.tiff]

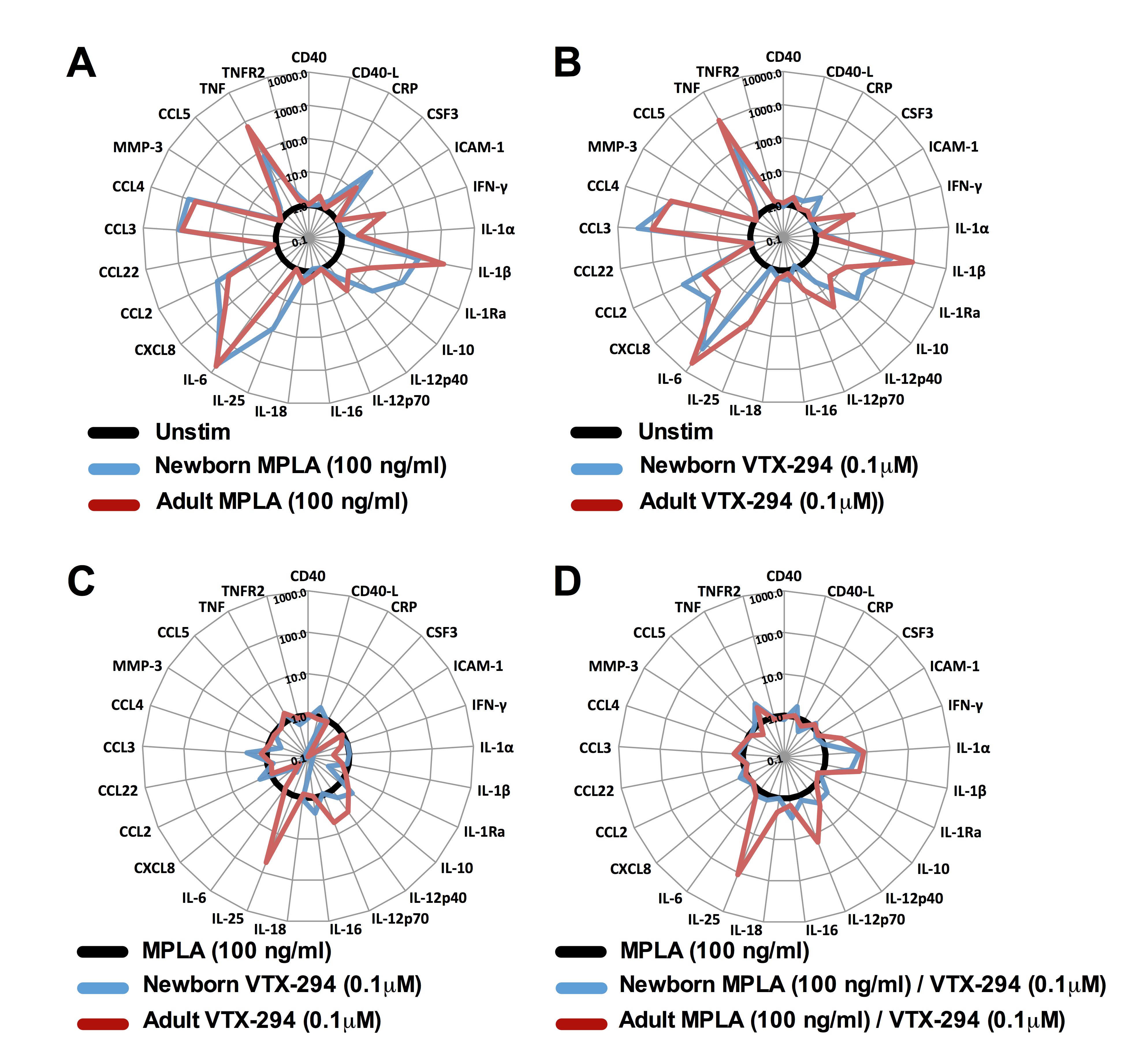

Supplement: Figure S4 — Comparison of MPLA, VTX-294 and combinatory MPLA-VTX-294-induced cytokines in newborn and adult blood. Human neonatal (blue) and adult (red) WB samples were cultured for 6 h. Cytokine responses of 25 analytes (pg/ml) are represented in radar plots showing fold-change of (A) un-stimulated vehicle to MPLA (TLR4, all 100 ng/ml), (B) un-stimulated vehicle to VTX-294 (TLR8, all 0.1 µM), (C) MPLA to VTX-294 (TLR8, all 0.1 µM) and (C) MPLA to MPLA with VTX-294, with a modified Human Inflammation MAP v. 1.6-antigen panel. Data are shown as mean ± SEM for n = 3. (TIFF) [file pone.0058164.s004.tiff]
