# Supplementary material for: The Ultra-Potent and Selective TLR8 Agonist VTX-294 Activates Human Newborn and Adult Leukocytes
Source: PLoS One. 2013 Mar 4;8(3):e58164. doi: 10.1371/journal.pone.0058164 (PMC3587566; doi:10.1371/journal.pone.0058164)
Supplement: Table S1 — (DOC) [file pone.0058164.s005.doc]

**Table S1**: Mean EC_50_ values for TLR agonist activation of HEK Cells and newborn and adult human whole blood (n = 4-9).

| **Agonist** | **MPLA EC_50_ (ng/ml)** | **R848 EC_50_ (μM)** | **CL075 EC_50_ (μM)** | **VTX-294 EC_50_ (μM)** |
| --- | --- | --- | --- | --- |
| TLR7 HEK Cell SEAP | N/A | 0.35 | 7.33 | 5.74 |
| TLR8 HEK Cell SEAP | N/A | 5.12 | 4.57 | 0.05 |
| Newborn WBA TNF | 14.57 | 1.50 | 13.90 | 0.90 |
| Adult WBA TNF | 12.59 | 1.04 | 12.00 | 0.27 |
| Newborn WBA IL-1β | 11.48 | 6.65 | 13.16 | 1.19 |
| Adult WBA IL-1β | 1.59 | 1.62 | 15.06 | 0.78 |

SEAP, secreted embryonic alkaline phosphatase; WBA, whole blood assay; IL, interleukin; TNF, Tumor necrosis factor; HEK, Human Embryonic Kidney, MPLA, monophosphoryl lipid A; N/A, not applicable.
