# Supplementary material for: The Ultra-Potent and Selective TLR8 Agonist VTX-294 Activates Human Newborn and Adult Leukocytes
Source: PLoS One. 2013 Mar 4;8(3):e58164. doi: 10.1371/journal.pone.0058164 (PMC3587566; doi:10.1371/journal.pone.0058164)
Supplement: Table S2 — (DOC) [file pone.0058164.s006.doc]

**Table S2**: Averaged maximum achieved cytokine values for TLR agonist activation of newborn and adult human whole blood (n = 4-9). Data shown with baseline cytokine levels in un-stimulated blood subtracted. Agonist concentrations are in μM, except for MPLA (ng/ml).

|  | **Agonist Conc.** | **Newborn WBA TNF (pg/ml)** | **Adult WBA TNF (pg/ml)** | **Newborn WBA IL-1β (pg/ml)** | **Adult WBA IL-1β (pg/ml)** |
| --- | --- | --- | --- | --- | --- |
| **MPLA (ng/ml)** | 0.1 | 0 | 128 | 0 | 5 |
|  | 0.3 | 11 | 33 | 0 | 27 |
|  | 1 | 29 | 215 | 24 | 95 |
|  | 10 | 642 | 3353 | 682 | 763 |
|  | 100 | 3665 | 9158 | 1628 | 3153 |
| **R848**  **(μM)** | 0.1 | 0 | 132 | 0 | 2 |
|  | 0.3 | 0 | 750 | 9 | 27 |
|  | 1 | 2164 | 6661 | 285 | 779 |
|  | 10 | 33609 | 13913 | 7442 | 4762 |
|  | 100 | 20117 | 9982 | 10822 | 4694 |
| **CL075**  **(μM)** | 0.1 | 31 | 19 | 0 | 0 |
|  | 0.3 | 0 | 0 | 0 | 4 |
|  | 1 | 207 | 100 | 58 | 67 |
|  | 10 | 11274 | 6001 | 4851 | 1160 |
|  | 100 | 32636 | 16206 | 19083 | 8462 |
| **VTX-294**  **(μM)** | 0.1 | 264 | 1905 | 159 | 333 |
|  | 0.3 | 10599 | 8522 | 2376 | 1274 |
|  | 1 | 22549 | 15219 | 7891 | 2882 |
|  | 10 | 43619 | 15664 | 18635 | 5065 |
|  | 100 | 29175 | 9612 | 13128 | 2796 |

WBA, whole blood assay; IL, interleukin; TNF, Tumor necrosis factor; MPLA, monophosphoryl lipid A.
